# Experimental Investigation of Color Reproduction Quality of Color 3D Printing Based on Colored Layer Features

**DOI:** 10.3390/molecules25122909

**Published:** 2020-06-24

**Authors:** Jiangping Yuan, Jieni Tian, Chen Chen, Guangxue Chen

**Affiliations:** 1 State Key Laboratory of Pulp and Paper Engineering, South China University of Technology, Guangzhou 510640, China; yuanjiangping2009@gmail.com (J.Y.); Tianjieni123@126.com (J.T.); chenchen_1224@126.com (C.C.); 2 Institute for Visualization and Data Analysis, Karlsruhe Institute of Technology, 76131 Karlsruhe, Germany

**Keywords:** 3D printing, color layer, printing sequence, color reproduction, quality evaluation

## Abstract

Color three-dimensional (3D) printing is an advanced 3D printing technique for reproducing colorful 3D objects, but it still has color accuracy issues. Plastic-based color 3D printing is a common color 3D printing process, and most factors affecting its color reproduction quality have been studied from printing materials to parameters in the fixed consecutive layers. In this work, and combined with variable stair thickness, the colored layer sequence in sliced layers of a specific 3D color test chart is deliberately changed to test the effects of colored layer features on its final color reproduction quality. Meanwhile, the colorimetric measurement and image acquisition of printed 3D color test charts are both achieved under standard conditions. Results clearly show that the chromatic aberration values and mean structural similarity (MSSIM) values of color samples have a significant correlation with the colored stair thickness, but both did not display a linear relationship. The correlation trends between colored layer sequence and the above two indexes are more localized to the colored stair thickness. Combined with color structural similarity (SSIM) maps analysis, a comprehensive discussion between colored layer features and color reproduction quality of color 3D printing is presented, providing key insights for developing further accurate numerical models.

## 1. Introduction

Color is an important attribute of realistic three-dimensional (3D) objects, and its accurate reproduction has always been a hot challenge in 3D printing [1]. Color 3D printing is a special stage in the evolution of the mainstream 3D printing processes, which provides more authentic color reproduction of functional 3D objects applied to various manufacturing industries [2,3]. Since 2014, color 3D printing has become more popular among the general public when the remarkable color 3D printers were released using different printing materials [4,5,6]. However, these color 3D printing processes do not really achieve accurate color reproduction, although they can print half a million colors [7,8].

In addition, the quality evaluation of 3D prints has always been the focus of scholars in the fields of functional materials and additive manufacturing [9,10]. Those quality evaluations consist of surface properties, structural strength, component characterization, and specific functional properties. Relative researchers also discussed the color attributes including color stability and color consistency when assessing the surface properties of different color 3D prints [11,12,13,14]. Color attributes of most color 3D prints were measured by colorimetric methods using various spectrophotometers [15]. These are easily understood by researchers in the fields of additive manufacturing and material development but are not enough for accurate color 3D printing. The parameters that affect color reproduction in color 3D printing are not only the coloring material itself, but also the assignments of the total printing materials in the printing process [16,17]. Moreover, the digital 3D model input including specific texture and slices is another key factor that needs to be reviewed, which is possible to fundamentally change the principle of color reproduction of 3D prints and rebuild its color reproduction evaluation system [18].

Currently, the color reproduction of color 3D printing has been faced with scarce online evaluation, although color vision has been maturely applied to quality information acquisition of 3D prints [19,20]. On the one hand, it is difficult to accurately achieve each photographed color layer during the printing process [21]; on the other hand, it lacks the corresponding recognized evaluation method [22]. An indirect solution is to convert a specific view surface of a 3D object into a corresponding image, and then quantify and characterize it using traditional image quality evaluation methods. Therefore, combining chroma evaluation and image-based difference evaluation, this study explored the effects of color layer features on the color reproduction of plastic-based color 3D printing processes. The color layer features consist of color layer sequence and color stair thickness. The color layer sequence is changed at the top color stair of our proposed 3D color test chart, and the color stair thickness is varied from the number of slicing layers. This will provide a unique assignment strategy for accurate color reproduction in color 3D printing to improve current sluggish color accuracy.

## 2. Results and Analysis

In this section, there are two parts: one is that the whiteness changes of six white blocks are used to verify whether its base thickness affects the color rendering of the upper stairs; the other is that the effects of color layer features on color reproduction of the entire 3D printed sample. For the graphical representations in the following subsections, the five printed layers are abbreviated to five layers in the figures. Next, ∆W1-00001 is the weight difference between sample 00001 and sample 1, then ∆E1-00001 is the color difference between sample 00001 and sample 1; other symbols in this section are all analogous in turn.

### 2.1. Whiteness Change Analysis of Printed White Blocks

**Figure 1:**
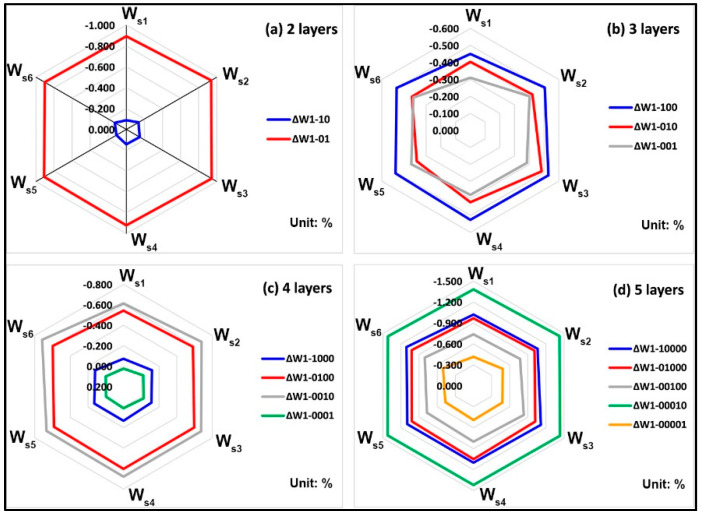
Whiteness changes between six white blocks with specific printed layers and blocks with one single layer (sample ID 1): (**a**) 2 layers; (**b**) 3 layers; (**c**) 4 layers; (**d**) 5 layers.

The whiteness values of all white blocks fluctuated slightly within the 71~72% range (relative whiteness). The whiteness change of six white blocks with specific printed layers is contrasted to that with one layer (sample ID 1 in Table 2), as shown in Figure 1. For all white blocks, comparing a stair with two layers, it can be observed that the whiteness of six different stairs in two samples (ID: 10, 01) is hardly changed as the colored stair thickness increases from Figure 1a. For each stair with three printed layers, the whiteness of all three samples (ID: 100, 010, 001) shows irregular variations, but all fluctuate only in a very small range. In Figure 1c, six stairs in four samples (ID: 1000, 0100, 0010, 0001) show regular concentric ring distribution without showing linear relationship. For each stair with five layers, the comparison results of ∆W1-00001 show that such slight fluctuations occur in the six colored stairs, while all other combinations were virtually unchanged in different stairs.

In summary, compared to that in sample 1, the whiteness of all other samples is barely increased with the number of printed layers, and no significant differences are also shown in the thickness comparisons of different colored stairs. Therefore, the white base added to assist the safety of the sample during the initial model design can be ignored in terms of its effect on the upper stairs including specific transparent layers and a color layer.

**Figure 2:**
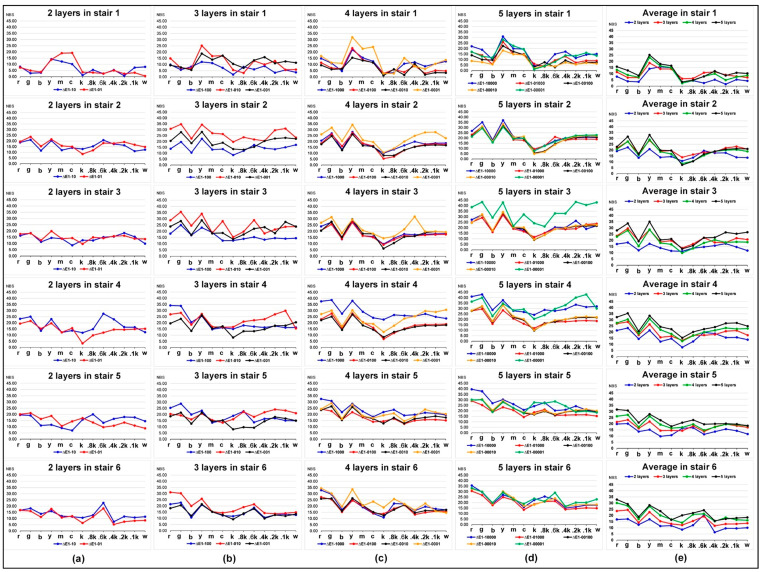
Chromatic aberration between samples with multiple printed layers and samples with one single layer (sample ID 1): (**a**) 2 layers; (**b**) 3 layers; (**c**) 4 layers; (**d**) 5 layers; (**e**) average.

As a result, the relative chromatic aberration of the color layers at the top is mostly small for the same colored stair with different printed layers. However, as the number of total printed layers increases, the color layer distribution order shows a similar decrease of its relative chromatic aberration value, but there is no linear correlation for those changes. The relative color difference of the samples in the middle order showed a more similar change trend, and the order of the color layer between them is not obvious on most color samples. In addition, an average comparison of the chromatic aberration for samples with different colored layer sequence in each stair is illustrated in Figure 2e. The average chromatic aberration values for 14 samples compared with the sample 1 are also clearly shown in Table 1. It is evident that the larger the stair number, the greater the chromatic aberration value, but no linear correlation.

### 2.2. Effects of Colored Stair Thickness on Color Difference of Printed Bars

The colored stair is always located at the top in all 15 samples and consists of six color stairs and seven neutral stairs above relative supported stairs filled with the same transparent layers. Its relative thickness is determined by the specific position of a color layer and lower total transparent layers. Meanwhile, these designed thicknesses are illustrated in Table 2, while it is more convenient to select the specific stair number (stair 6, stair 5, stair 4, stair 3, stair 2, stair 1) when analyzing.

In Figure 2, the relative color difference of colored stair is compared to the stair with only one layer (sample ID 1 in Table 2). For each stair with two layers, except for stair 6, all other colored stairs showed that the chromatic aberration of the color layer at the bottom is greater than at the top, while that of the neutral layers showed irregular up and down fluctuations. Samples with three layers were similar to samples with two layers, but what makes it different is the stair 5. For each stair with four layers, the irregular color sample is just the stair 4. Meanwhile, the samples in the middle order showed similar trends in the color layers and the neutral layers for most stairs. For each stair with five layers, except for the stair 3, none of the other stairs showed an increase in the chromatic aberration of the color layer at the bottom compared to the top. However, no linear correlation was found for the position of the color layers and neutral layers among six stairs.

**Figure 3:**
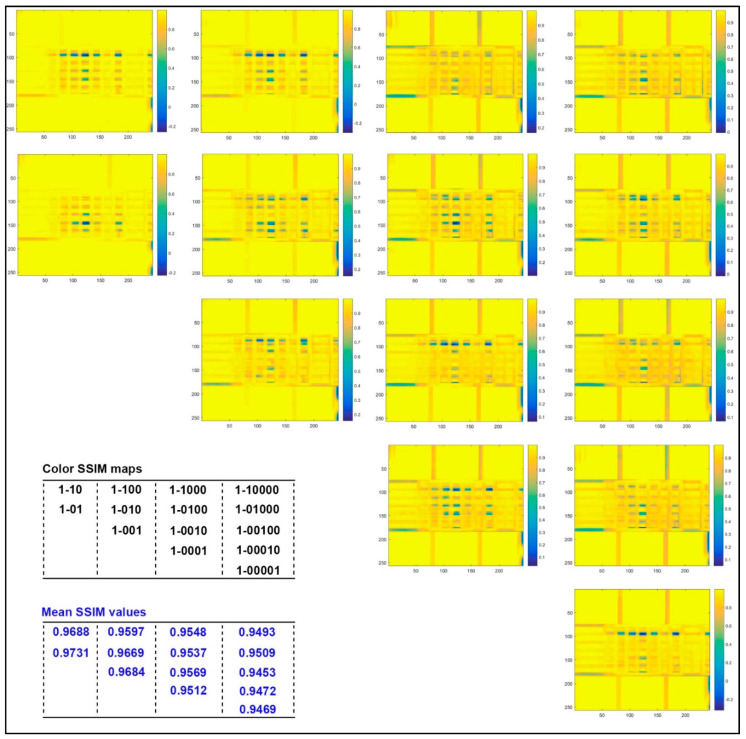
Color structural similarity (SSIM) maps and mean structural similarity (MSSIM) values for captured images of 3D color test charts compared to the sample with one single layer (sample 1).

Figure 3 shows the MSSIM values and relative similarity maps for captured images of 3D color test charts with four kinds of specific printed layers compared to those with the single layer (sample ID 1 in Table 2). It can be found that the corresponding MSSIM value of the acquired image decreases as its number of print layers in the specific stair increases, but there is no linear correlation overall. With the same number of printed layers in each stair, the MSSIM value increases as the color layer and neutral color layer are located further down. Meanwhile, as the number of printed layers per specific stair increases, the above phenomenon changes from the global to local adjacent printed layers. From the similarity maps with marked color bars, the apparent variance of each colored stair occurs mainly at the junction of stair 6 and stair 5, at the junction of stair 3 and stair 2, and at the junction of stair 2 and stair 1. However, areas of minor variation (orange areas) also showed a slight decreased similarity with increasing number of printed layers per stair, even though it did not show a direct correlation with the stair number. For the color distribution in the differential region, samples with different printed layers were found in both color and neutral samples. For example, relative color samples are y and c, but the neutral samples are k and 0.8 k.

### 2.3. Effects of Colored Layer Sequence on Color Difference of Color Patches

**Figure 4:**
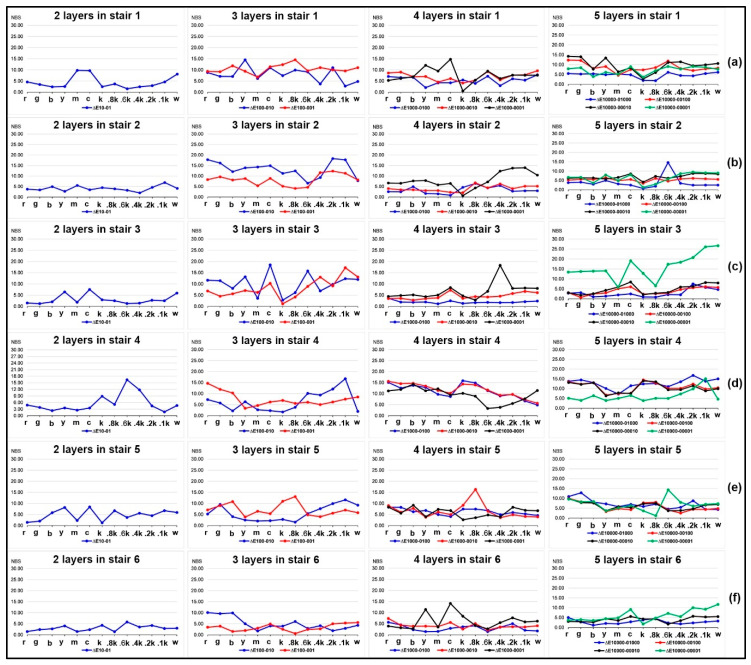
Chromatic aberration of samples for each other comparison: (**a**) stair 1; (**b**) stair 2; (**c**) stair 3; (**d**) stair 4; (**e**) stair 5; (**f**) stair 6.

In Figure 4, it can be seen that the relative chromatic aberration of tested 3D color test charts with the same number of printed layers is much smaller than that of the single layer sample in Figure 2. For the four comparisons in colored stair 1, under the same number of printed layers, the effect of the printing order of the colored layers on its relative color difference is that the chromatic aberration is mostly greater when the position is lower, but this phenomenon did not show linear correlation. The regular quantitative correlation between colored layer sequence and chromatic aberration cannot be found from the colored stair 2. In terms of colored stair 3, a specific correlation between the colored layer sequence and the relative chromatic aberration appeared under the same number of printed layers, except for the samples with three layers. Meanwhile, the correlation consistency for all color bars were obvious while the neutral color bars were not as pronounced. The correlation trends of colored stair 4 and stair 5 were similar, and both globally failed to change linearly with the printing sequence. For the four comparisons in colored stair 6, the relative chromatic aberration of each colored layer at the bottom is greater than that at the top under the same number of printed layers, except for the stairs with three layers. However, the overall color variation of all colored stair 6 does not strictly follow the coloring layer sequence. As a result, all numbered stairs did not globally form a regular quantifiable correlation between the colored layer sequence and its relative chromatic aberration, which requires further studies.

**Figure 5:**
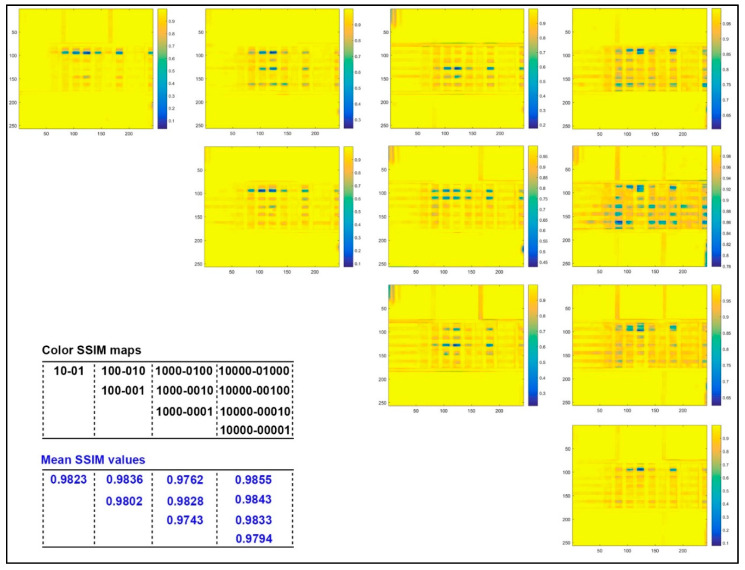
Color SSIM maps and MSSIM values for captured images of 3D color test charts compared with each other based on the same printing layers.

In Figure 5, the MSSIM values and color similarity maps for captured images of 3D color test charts with different colored layer sequences were illustrated under the same printed layers. It can be found that the lower the colored layer located in the stair with same printed layers, the smaller its MSSIM value, but again no linear correlation is presented here. For the color similarity maps with two layers, the significant difference in the 3D color test charts with different colored layer sequences occurred at the junction of colored stair 6 and stair 5. There are two significant difference areas in the stairs with three layers: one is the junction of colored stair 6 and stair 5; the other is the junction of colored stair 4 and stair 3. For the 3D color test charts with four layers, samples with different colored layer sequences showed obvious differences at the junction of colored stair 5 and stair 4, as well as the junction of colored stair 4 and stair 3. There are five colored layer sequences in the 3D color test charts with five layers. Meanwhile, two significant differences for four comparisons occurred at the junction of colored stair 6 and stair 5, as well as at the junction of colored stair 2 and stair 1. However, a strange phenomenon is that the similarity map of the colored layer at the bottom showed fewer areas of significant difference while its MSSIM value was the smallest. Figure 5 did not show all similarity comparisons for samples with five layers, because we believed that the current four comparisons of all other samples with the colored layer at the top provide sufficient correlational information to explore the effect of colored layer sequence.

The color difference analysis of 14 samples compared with sample 1 simulates the color reproduction difference between the new coloring method and the traditional method in the color 3D printing process. The color difference analysis of the samples with the same printing layers is mainly to explore the effect of the colored layer sequence. From the results of color difference analysis in Figure 2 and Figure 4, the current quantitative correlation has not yet formed an effective numerical model, but its impact on color reproduction quality is still worth constructing a predictive model. From the results of MSSIM values analysis in Figure 3 and Figure 5, the computational MSSIM value of each sample is still very high. The change of MSSIM values of samples with current colored layer features is not obvious, but the visual difference in relative color SSIM maps can be used as a reference for assessing the color reproduction quality of 3D printed parts.

## 3. Experimental

**Figure 6:**
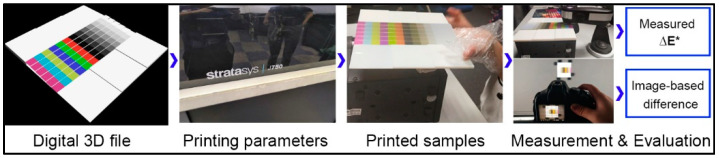
The flowchart of our experiment.

The color reproduction quality of color 3D prints is easily affected by the input 3D model and selected printing parameters. In this study, we focus on the effects of color layer features on color reproduction quality of 3D printed color objects, such as color layer sequence and colored stair thickness. The flowchart of our experiment is shown in Figure 6. It consists of five parts: digital 3D model design, printing parameters setting, printed samples preprocess, measurements, and objective evaluations. The Stratasys J750 3D printer is used to print all tested 3D models with the same printing materials and parameter settings. The printing materials consist of the Vero-Pure-White, Vero-Black, Vero-Clear, Vero-Magenta, Vero-Cyan, and Vero-Yellow. The printing parameters mainly consist of the color texture mapping, slicing parameters, and material arrangement. The minimum printing accuracy is 0.2 mm for each layer. The printed samples preprocess is to remove support materials of 3D prints. The color texture of a specific 3D model is automatically mapped to the coloring material system based on its own calibration principles. Theoretically, this is not the optimal color mapping solution, but indeed the most common method in industrial applications, which is the key issue for the current color 3D printing color reproduction quality to be improved.

### 3.1. Designing the 3D Color Test Charts

**Figure 7:**
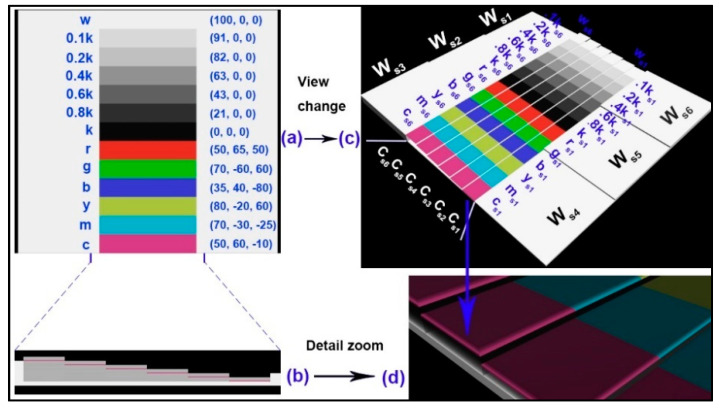
A three-dimensional (3D) color test chart: (**a**) top view; (**b**) local side view; (**c**) global view with relative marks; (**d**) local details of stairs.

In graphics printing, developed color test charts are plentiful for various printing substrates, but there are so few in color 3D printing. Thus, we designed a special 3D color test chart using the 3D Studio Max software (Autodesk, San Rafael, CA, USA), as shown in Figure 7. The 3D color test chart consists of six color bars, seven neutral color bars, and six white blocks. In color bar regions, primary colors with acronyms are displayed in order from one end to the middle, such as c (cyan), m (magenta), y (yellow), b (blue), g (green), and r (red). In neutral color bar regions, color samples are shown in sequence from the middle to the other end, such as k, 0.8k, 0.6k, 0.4k, 0.2k, 0.1k, and w. Here, the k means the black color, the 0.8k means a gray (80% black) color, and the w means a white (0% black) color. The original L^*^, a^*^, and b^*^ values of each bar are shown in Figure 7a. Color bars and neutral color bars are assigned to corresponding identical stairs including stair 6 (s6), stair 5 (s5), stair 4 (s4), stair 3 (s3), stair 2 (s2), and stair 1 (s1), as shown in Figure 7c. For the colored stairs with 13 color bars, the C_s6_ means that the color layer is placed in the stair 6 in which there is a total of six specific stairs above the base; other markers are analogous in turn. Similarly, the W_s6_ corresponds to a white block in the stair6 with six same white stairs. In each stair, the specific number of printed layers is easily defined by the J750 3D printer (Stratasys, Eden Prairie, MN, USA). Here, one printed layer thickness is 0.2 mm. Meanwhile, each white block shows the same height as the corresponding colored stair underneath, respectively.

Due to the variables including the number of printed layers and the color layer sequence in each stair, we designed 15 kinds of samples based on this 3D color test chart, as shown in Table 2. This table also shows the detailed print thickness for each stair in all test charts. The sample ID is named by the color layer sequence in the relative upper stair. For example, the sample ID 00001 means that its color layer (using the symbol 1) is located at the bottom printing layer of the stair with five printing layers, while the symbol 0 means that this layer should be printed with transparent ink. For the color bar, its entire printing thickness is defined by the sample that the color layer is located at the top place, such as the sample: 1, 10, 100, 1000, 10,000. Other cases show the positional altitude of the colored layer in all specific stairs. There are five kinds of specific layers in each stair, such as 1 layer, 2 layers, 3 layers, 4 layers, and 5 layers. Meanwhile, all stairs in each 3D color test chart are designed for only one of the above printed layers. In addition, there are five color layer sequences for the stair with five printed layers, such as sample: 10000, 01000, 00100, 00010, 00001; other cases are analogous in turn. It should be explained that the base in Table 2 is not what we want, but a guarantee that every sample is printed intact. The more layers printed, the thicker the whole model becomes, then the required base becomes thinner. Since all bases are printed with opaque white material (Vero-Pure-White ink), the thickness of the bases theoretically does not affect the color reproduction of its upper test samples; we also conducted experimental verification as shown in Section 2.1.

### 3.2. Properties Measurement of Printed 3D Color Test Charts 

In this study, two properties of all printed samples were measured with colorimetric values of bars and whiteness of blocks. The colorimetric values of printed 3D samples are measured by the calibrated i1 Basic Pro2 (X-Rite, Grand Rapids, MI, USA), and recorded with the L*, a*, and b* values. The measurement method of this spectrophotometer is based on the ISO 13655 M1 with the D50. Each bar is measured three times and analyzed with its average. Meanwhile, the whiteness of six white blocks is measured by the Color-Touch @ PC CTP-ISO (Technidyne, New Albany, IN, USA) with the D65 and 2° view-field. In addition, this device can provide four different calibrated light sources or two user defined sources. Its color data can be calculated with six color spaces, 10 illuminants, and two observers, which allow customer specific data presentations.

### 3.3. Image-based Difference Assessment of Printed 3D Color Test Charts

**Figure 8:**
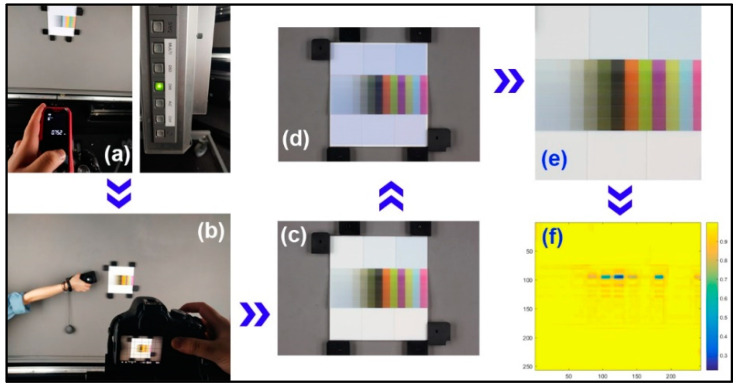
Image-based difference assessment: (**a**) condition configuration; (**b**) high-definition (HD) camera calibration; (**c**) acquired image pretreatment; (**d**) color sample recognition; (**e**) color sample segmentation; (**f**) color structural similarity (SSIM) map.

Although measurement of the above-mentioned properties is good for the offline environment, the image-based difference is suitable for online measurement and color reproduction evaluation of 3D printed products. The flow chart of image-based difference assessment is shown in Figure 8. In the object imaging system, the light source is the D65 and the shooting distance is 0.752 m, as shown in Figure 8a. The high-definition (HD) camera is a Canon EOS 500D with a 0.35 mm/1.1 ft-∞ lens. To acquire the accurate color of 3D prints, this HD camera is calibrated with an inherent white reference and 2D color chart, which provides an acquired color difference within 2 NBS. The original acquired image is renamed and horizontally calibrated by Adobe Photoshop software. The next step is to create a sampling window of the same size to recognize the 3D color test chart in the whole image (see Figure 8d). Then the corresponding color samples are manually segmented one by one. At last, a specific 3D color test chart is compared with another 3D color test chart, and the relative similarity map and its mean structural similarity (MSSIM) index are achieved by our own MATLAB code.

### 3.4. Data Analysis 

In this study, color difference and MSSIM index will be counted by the above-mentioned original measurements. The CIEDE76 formula is used to calculate the relative color difference of each color point on 3D printed color bars, as shown in Equation (1). The structural similarity (SSIM) index and MSSIM index of each acquired image are calculated by Equation (2) and Equation (3).

(1) $$\Delta E_{76}^{*}=\sqrt{{\left(L_{x}^{*}-L_{1}^{*}\right)}^{2}+{\left(a_{x}^{*}-a_{1}^{*}\right)}^{2}+{\left(b_{x}^{*}-b_{1}^{*}\right)}^{2}}$$

where ΔE*_76_ is the chromatic aberration computed by the CIEDE76 definition, and its unit is the NBS. The L*_1_, a*_1_, and b^*^_1_ is the measured color data of each color patch with only the first color layer, and the L*_x_, a*_x_, and b^*^_x_ is that color layer in the x layer, where x is a positive integer selected from 2 to 5.

The structural similarity (SSIM) index is shown below with the lite form for universal image quality assessment, and it is further modified as the MSSIM index that is a better image quality index using local window statistics [23].

(2) $$SSIM\left(x,y\right)=\frac{\left(2\mu_{x}\mu_{y}+C_{1}\right)\left(2\sigma_{xy}+C_{2}\right)}{\left(\mu_{x}^{2}+\mu_{y}^{2}+C_{1}\right)\left(\sigma_{x}^{2}+\sigma_{y}^{2}+C_{2}\right)}$$

where *μ_x_* and *μ_y_* is the mean luminance intensity of ***x*** signal and ***y*** signal, respectively; *σ_x_* and *σ_y_* is the relative standard deviation; *σ_xy_* is the covariance of *σ_x_* and *σ_y_*; *C_n_*=(K_n_*L) ^*2^; *L* is the dynamic range of the pixel values (255 for 8-bit grayscale images)*; C_1_* and *C_2_* are the configurable constants. In this study, the *K_1_* in *C_1_* and *K_2_* in C_2_ are 0.01 and 0.03.

(3) $$MSSIM\left(X,Y\right)=\frac{1}{M}\overset{M}{\underset{j}{\sum }}SSIM\left(x_{j},y_{j}\right)$$

where **X** and **Y** are the reference and the varied images, respectively; *x_j_* and *y_j_* are the image contents at the *j* local window; and *M* is the number of local windows of the image. In addition, to illustrate the vivid difference map, we further optimized its grayscale map to a red-green-blue (RGB) color map achieved by our own MATLAB code.

## 4. Discussion and Conclusions

Based on the proposed unique 3D color test chart, 15 samples with specific colored layer features were printed by the plastic-based color 3D printer. This 3D color test chart not only can detect the color reproduction in the horizontal direction of the printed layer, but also can reflect the color accuracy in the vertical direction of the colored layer, which is theoretically more suitable for the color reproduction quality evaluation for any color 3D printers. This paper studied two important variables of colored layer features that affect the color accuracy of color 3D prints. One is the colored stair thickness and the other is the colored layer sequence at the top stair. For current color 3D printers, the single colored layer is usually set as the top layer, while this colored layer sequence proved not to be the optimal for the accurate reproduction of 2.5D oil painting and 3D topographic map. In addition, the colored layer sequences of the total 3D object are also affected by the thickness of each sliced layer. Therefore, the thickness of specific colored stairs including the single colored layer and transparent layers on the color reproduction were also tested by a certain number of sliced layers.

In Section 2, the first experiment showed that the colored stair thickness did affect the color reproduction quality of color 3D printed samples. The significant trend that the relative chromatic aberration of the colored layer at the top is smaller than that at the bottom, became localized as the number of print layers increases. However, this effect cannot be ignored as before, although we currently do not find a significant quantitative correlation. Moreover, the results in Figure 2e showed that similar trends can be achieved by further improvement of the printing accuracy, providing a further guide to color management of any material-jetting color 3D printers. In addition, the comparison of MSSIM values of colored layers at the top and the bottom was opposite to their corresponding chromatic aberrations. Combining with the color SSIM maps, it can be explained that the calculated weight of structural differences is bigger for their acquired images of color 3D prints. This trend was varied from the primary colors, but also showed a specific relationship with its structural thickness in each colored stair.

Similarly, the second experiment presented certain localized quantitative relationships among samples with different colored layer sequence and the same printed layers in each stair. These significant correlation trends become more unstable as the number of printed layers in each stair increases. A possible core reason is that the colored layer sequence also causes the print quality of the colored stair to change in the vertical direction. For example, the difference between printing the color layer on the transparent layer and printing the transparent layer on the color layer is that the substrate interface is different, which is affected by the specific characteristics of color resin injection and transparent resin injection. In addition, the MSSIM values of samples with different printing order and the same stair thickness became smaller as the colored layer located lower, but they did not show linear correlation. Meanwhile, there were two significant difference regions for the above-mentioned samples when comparing their color similarity maps, but these regions also obviously changed with the colored layer position.

When evaluating the color reproduction quality of color 3D prints, this paper focused on two objective scales based on colorimetric measurement and image-based computation to verify the applicability of the proposed 3D color test chart as the practical tool. Colorimetric measurement was widely used for evaluating the surface color of 3D printed objects, as an essential method to offline measure and calibrate the color 3D printer. The limitation is that current color difference formulas in 2D printing are not perfect for human visual perception among all colors and are roughly applied to color 3D prints without empirical evidence and modified numerical models. For the color image similarity evaluation, the image structural analysis is essential for images acquired by color vision systems. The original images obtained are all of the same size and resolution, but the target area split within each original image is difficult to meet such requirements for accurate quantitative association. In addition, efficient imaging of the entire print layer during the printing process still is a problem to be solved for color 3D printers. One way is the integrated method including segmentation and fusion algorithms for image acquisition from multiple angles. The unique bottom-up stair design proposed in this study provides a strategy to statically simulate the surface information acquisition of each printed layer during the printing process. In addition, the hue-angle index and the improved color-image-difference (iCID) metric index are used for the color image quality evaluation [24].

The main limitations of our current experimental results are further summarized here. Firstly, the minimum printing resolution of the current printed sample is 0.2 mm rather than 0.1 mm, which increased the overall height of the sample with five printed layers in a colored stair. The colored layers’ features are sensitive to the printing resolution of colored layer that become more practical for numerical modeling. Secondly, the color purity of the current primary color resin and black resin still needs to be improved, such as the yellow ink, cyan ink, and black ink. All these resins assignments determine the boundaries of the output color gamut to guide the operator or control system achieving better color matching. Next, the color difference formula used in this paper is the CIEDE76, which is the simplest evaluation tool for two color points or patches, while its inherent issue is not accurately corresponding to the human visual perception. The optimal selection of the chromatic aberration formula also needs to take into account the corresponding subjective scales. Finally, the designed segmentation mask guarantees the same size and resolution for segmented areas, but its precise positioning in each original acquired image is a challenge resulting in a greater weight of the image structure parameters. Meanwhile, the constant array [*K_1_*, *K_2_*] used in this experiment adopted the default values [*0.01*,*0,03*], but this setting was not necessarily optimal for the 3D object images.

Integrated color rendering mechanism between the color layer and the filled layer is a new direction for the current color 3D printing color reproduction quality improvement. Namely, color 3D printing is no longer absolutely printing a colored layer at the top, but rather an adaptive colored layer sequence assignment that takes into account the overall or local characteristics of the 3D model. Since color 3D printing is too expensive, this study only explored the colored layer sequence in the top color stairs, and firstly verified that this objective effect does exist for color 3D prints, although we did not consider the case in the internal stairs. The main contribution of this article is to provide a comprehensive evaluation strategy for color reproduction quality of color 3D prints, and to share inspiration for the key issues in the color reproduction quality evaluation system.

## Figures and Tables

**Table 1:** The average color difference of all stairs with relative printing layers.

Unit: NBS		r	g	b	y	m	c	k	0.8k	0.6k	0.4k	0.2k	0.1k	w
Stair 1	2 layers	7.73	3.90	3.50	14.00	15.65	14.70	2.46	4.44	2.48	5.23	1.58	5.30	4.25
	3 layers	11.40	6.89	6.93	18.72	13.95	13.85	6.02	6.20	10.83	11.59	9.09	7.97	7.03
	4 layers	13.16	8.98	7.23	23.13	16.31	14.94	2.41	4.59	7.85	10.06	4.92	7.47	8.58
	5 layers	15.71	12.68	8.55	25.20	17.90	16.47	3.40	4.91	7.84	12.10	8.63	10.81	10.28
Stair 2	2 layers	19.12	22.36	13.44	20.89	13.84	14.50	10.70	13.56	19.47	17.56	17.68	13.87	13.56
	3 layers	21.72	27.45	17.21	28.31	18.99	19.63	13.90	16.23	18.07	18.45	21.87	23.08	20.95
	4 layers	20.74	27.38	15.55	28.98	18.59	16.75	8.53	10.64	15.50	18.96	19.94	20.38	18.94
	5 layers	22.89	31.53	16.64	32.99	19.86	19.67	7.36	10.40	16.62	18.99	20.74	21.23	21.07
Stair 3	2 layers	17.00	18.28	11.96	17.24	13.89	11.50	11.18	13.71	14.70	15.74	17.39	14.66	11.77
	3 layers	23.63	29.61	19.52	28.62	18.67	19.78	13.85	17.05	22.14	18.30	18.18	21.75	20.69
	4 layers	22.99	28.01	15.37	28.69	17.94	16.78	9.89	13.51	17.87	20.36	18.31	18.75	18.56
	5 layers	27.97	33.65	19.27	35.00	20.36	21.12	13.12	15.73	22.06	22.27	26.41	25.28	26.55
Stair 4	2 layers	21.33	23.32	14.42	21.38	12.25	14.68	7.58	12.37	19.78	18.73	15.50	15.68	13.80
	3 layers	26.89	28.39	17.53	26.36	15.70	16.62	13.26	17.43	17.46	18.01	20.70	21.25	17.42
	4 layers	27.49	30.41	18.66	30.71	21.94	18.44	12.65	17.14	19.93	21.42	23.38	22.54	22.89
	5 layers	32.23	35.35	20.74	33.38	24.35	22.34	15.30	20.14	22.43	24.08	27.19	27.50	24.87
Stair 5	2 layers	19.81	20.12	13.68	15.18	9.77	10.68	16.39	16.76	11.41	13.63	15.69	14.28	11.64
	3 layers	21.46	23.22	16.23	21.81	14.42	14.64	14.36	18.19	13.69	17.65	19.71	18.40	17.05
	4 layers	26.14	27.26	17.33	26.06	19.34	16.40	17.07	19.60	14.72	17.32	19.62	19.18	18.12
	5 layers	31.61	30.82	20.97	27.86	22.89	17.90	20.90	23.09	19.60	19.93	20.10	19.71	18.41
Stair 6	2 layers	16.76	17.11	12.50	16.88	11.39	11.73	8.51	12.05	20.42	6.34	9.56	9.53	10.02
	3 layers	23.57	24.53	14.26	22.73	15.30	13.18	12.18	15.50	19.22	11.62	13.09	13.12	13.70
	4 layers	30.12	27.83	17.05	27.37	20.06	16.84	14.05	20.78	21.34	15.26	18.27	16.46	16.06
	5 layers	32.88	29.20	18.90	28.31	23.54	16.44	20.11	21.98	24.22	15.63	17.21	17.90	18.39

**Table 2:** The printing thickness for each stair in all 3D color test charts.

Unit: mm	White Blocks						Color Bars						
Sample ID	W_s1_	W_s2_	W_s3_	W_s4_	W_s5_	W_s6_	C_s1_	C_s2_	C_s3_	C_s4_	C_s5_	C_s6_	Base *
1	0.0	0.2	0.4	0.6	0.8	1.0	0.2	0.4	0.6	0.8	1.0	1.2	2.0
10	0.2	0.6	1.0	1.4	1.8	2.2	0.4	0.8	1.2	1.6	2.0	2.4	2.0
01	0.0	0.4	0.8	1.2	1.6	2.0	0.2	0.6	1.0	1.4	1.8	2.2	2.0
100	0.4	1.0	1.6	2.2	2.8	3.4	0.6	1.2	1.8	2.4	3.0	3.6	2.0
010	0.2	0.8	1.4	2.0	2.6	3.2	0.4	1.0	1.6	2.2	2.8	3.4	2.0
001	0.0	0.6	1.2	1.8	2.4	3.0	0.2	0.8	1.4	2.0	2.6	3.2	2.0
1000	0.6	1.4	2.2	3.0	3.8	4.6	0.8	1.6	2.4	3.2	4.0	4.8	1.0
0100	0.4	1.2	2	2.8	3.6	4.4	0.6	1.4	2.2	3.0	3.8	4.6	1.0
0010	0.2	1.0	1.8	2.6	3.4	4.2	0.4	1.2	2.0	2.8	3.6	4.4	1.0
0001	0.0	0.8	1.6	2.4	3.2	4.0	0.2	1.0	1.8	2.6	3.4	4.2	1.0
10000	0.8	1.8	2.8	3.8	4.8	5.8	1.0	2.0	3.0	4.0	5.0	6.0	1.0
01000	0.6	1.6	2.6	3.6	4.6	5.6	0.8	1.8	2.8	3.8	4.8	5.8	1.0
00100	0.4	1.4	2.4	3.4	4.4	5.4	0.6	1.6	2.6	3.6	4.6	5.6	1.0
00010	0.2	1.2	2.2	3.2	4.2	5.2	0.4	1.4	2.4	3.4	4.4	5.4	1.0
00001	0.0	1.0	2.0	3.0	4.0	5.0	0.2	1.2	2.2	3.2	4.2	5.2	1.0

* This base is essential to ensure that each printed model does not break during printing and measuring.
